# Granulicatella bacteraemia in children: two cases and review of the literature

**DOI:** 10.1186/1471-2431-13-61

**Published:** 2013-04-22

**Authors:** Maia De Luca, Donato Amodio, Sara Chiurchiù, Maria Assunta Castelluzzo, Gabriele Rinelli, Paola Bernaschi, Francesca Ippolita Calò Carducci, Patrizia D’Argenio

**Affiliations:** 1 Unit of Immunology and Infectious Disease, University Hospital Pediatric Department, Bambino Gesù Children’s Hospital, Piazza Sant’Onofrio 4, Rome, Italy; 2 Unit of Cardiology, Bambino Gesù Children’s Hospital, Piazza Sant’Onofrio 4, Rome, Italy; 3 Unit of Microbiology, Department of Laboratories, Bambino Gesù Children’s Hospital, Piazza Sant’Onofrio 4, Rome, Italy

**Keywords:** Granulicatella, Endocarditis, Bacteremia, NVS, Treatment, Meropenem, Paediatrics

## Abstract

**Background:**

Granulicatella spp. is a fastidious bacteria responsible for bacteremia and endocarditis which are fatal in about 20% of the cases. These severe infections are uncommon in children under 17 years of age and have proven extremely difficult to treat.

**Cases presentation:**

We report a brief review of the literature and two cases of NVS bacteremia by Granulicatella complicated by infective endocarditis (IE). The first one is that of a 7-year-old Caucasian female with Shone syndrome and IE involving the pulmonary valve homograft, confirmed by echocardiography. The second case is that of a 5-year-old Caucasian male. In this patient echocardiogram was negative for signs of IE; however, a “possible” IE was suspected on the basis of a cardiac catheterization 3 weeks before the onset of fever. Since in both our patients clinical failure of first line antibiotic treatment was observed, we used a combination of meropenem with another anti-streptococcal drug with excellent results.

**Conclusion:**

In Granulicatella bacteremia in the pediatric population, combination antimicrobial therapy including meropenem should be considered as a second line treatment in non-responding patients.

## Background

Granulicatella is a fastidious Gram-positive nutritionally variant streptococcus (NVS) that is classified in two different genera: Abiotrophia which includes only A*. defectiva* and the genus Granulicatella which comprises three species (G. *adiacens*, G. *elegans* and G. *balaenopterae*). Since these organisms require pyridoxal or thiol group supplementation for growth, their isolation may be difficult. Granulicatella *spp* is part of the normal flora of the oral cavity, the genitourinary tract, and the intestinal tract. Although NVS are found as part of normal flora of the upper respiratory, urogenital, and gastrointestinal tracts, their ability to cause clinically significant disease has been increasingly recognized. The most frequent clinical syndromes caused by NVS are endocarditis and bacteraemia [1] but these microorganisms have been implicated also in several others infections such as central nervous system infections [2], sinusitis, otitis media, prostatitis, cholangitis, arthritis [3]. In vivo and in vitro antimicrobial susceptibility tests suggest that peniclillin plus aminoglycoside or vancomycin alone should be considered as therapeutically equivalent [4], although antibiotic resistance has been described for both these drugs [5,6].

IE is uncommon in children under 17 years of age, but it is a cause of significant morbidity. Notably, 90% of IE cases occur in individuals who have structural heart disease, usually congenital. However, even children with normal hearts, could be at risk of IE due to invasive procedures such as bronchoscopy, tonsillectomy etc. [7]. The clinical presentation of IE is usually indolent with prolonged low grade fever associated with non-specific symptoms such as myalgia, arthralgia, headache and generalize malaise whilst classical signs of IE (e.g. Roth spots, Osler nodes) are very rare in children [8]. Mortality rate ranges from 4% to 18% and complications include valvular insufficiency, congestive heart failure, embolization, mycotic aneurism, etc. Cardiovascular surgery may be life-saving in these patients, but decision for surgical intervention must be individualized [7,8].

NVS have been implicated in approximately 5% of cases of bacterial endocarditis in the adult population [4] and carry a worse prognosis compared to infection with other streptococci [9]. In patients with endocarditis caused by *Granulicatella*, classic endocarditis signs such as digital clubbing, Osler nodes and petechiae are rare. Pre-existing valvular pathology is a frequent predisposing factor. Because of the difficulties in isolating this organism its role as a pathogen in endocarditis could be underestimated. Consequently, some cases of culture-negative endocarditis could be attributed to this microorganism [2].

Here, we report two cases of paediatric NVS bacteraemia, one of which with confirmed heart localization and review of the literature.

## Case presentation N.1

The first case concerns a 7-year-old Caucasian female with Shone syndrome (characterized by coartation of aorta, mitral stenosis and subvalvular aortic stenosis). At the age of 1 month she underwent a coartectomy and 6 years later she underwent a Ross-Konno procedure (aortic valve replacement with a pulmonary autograft and pulmonary valve replacement with a homograft conduit) plus mitral valvuloplasty. The last echocardiogram, performed 1 month before admission to our hospital, showed a subtotal obstruction of the conduit.

She was admitted to our hospital with a history of 2-weeks of nocturnal fever, treated at home with ceftriaxone without success. The patient appeared acutely ill; clinical examination revealed a 2-3/6 systolic murmur and hepatosplenomegaly. A complete blood count revealed anemia (Hb 10.3 g/dl), white blood cells count 15,760/ml with 84.2% neutrophils and 11.7% lymphocytes, CRP 4.86 mg/dl. Transthoracic echocardiogram was negative for signs of IE. The child had fever to 39°C. Vancomycin and gentamicin were started, with subsequent defervescence. Blood samples were taken for cultures. After 4 days of therapy, fever recurred and was associated with shaking chills. Gram-positive cocci in short chains were isolated from blood cultures, but there was very scant growth on routine bacterial culture media. Applied Biosystems 3130 and 3130xl Genetic Analyzers identified the organism as *G. adiacens*. Antibiotic susceptibility tests (AST) were not available. A repeat echocardiogram demostrated a filamentous (10 × 2 mm) on the homograft. Antibiotic therapy was changed to vancomycin plus meropenem.

After four weeks, therapy was switched to oral amoxicillin and clavulanic acid. The patient began to complain of right-side abdominal and chest pain. Suspecting pulmonary embolism, a lung computer tomography scan was performed. This revealed a pyramid-shaped area of pleural-based consolidation in the posterior segment of the right lower lobe. No significant filling defects of the pulmonary arteries and their major branches were evident. Intravenous treatment with vancomycin plus meropenem was restarted and continued for additional two weeks; low molecular weight heparin was prescribed. However, due to a persistent dysfunction of the conduct, it was replaced with a pulmonary valved homograft. Cultures of prosthetic material were negative. Six months later the patient was doing well and had negative blood cultures.

## Case presentation N.2

A 5-year-old Caucasian male was brought to our hospital with a 2-month history of fever and associated diarrhoea and leg pain. His past medical history was significant for infundibular pulmonary stenosis. Cardiac catheterization had been performed 3 weeks before the onset of fever. At admission, he presented general malaise, asthenia, anorexia, pale skin and a systolic murmur. Laboratory tests revealed a white blood count of 8,370/ml with 70% neutrophils and CRP 2.99 mg/dl. Blood cultures were obtained and empiric antibiotic treatment with cefotaxime and gentamicin was started. Transthoracic and transesophageal echocardiograms revealed no vegetations and chest x-ray and abdominal ultrasound were negative. Two blood cultures taken 24 hours apart isolated *G. Adiacens*, susceptible to cephalosporins, vancomycin, tetracycline, cloramphenicol, linezolid and resistant to penicillin. Despite antimicrobial therapy, the patient’s fever persisted and five days after admission two blood cultures were again positive for *G. adiacens* that was now resistant to beta-lactams, tetracycline, cloramphenicol, linezolid, susceptible to vancomycin, clindamycin and levofloxacin (MIC 1.5 mcg/ml, 0.125 mcg/ml and 0.38 mcg/ml respectively). Meropenem was not tested. Therapy was changed to ciprofloxacin (10 mg/kg q12) and meropenem (30 mg/kg q8). Repeated transthoracic and transesophageal echocardiograms were again unchanged from the patient’s baseline; nonetheless, these findings led us to consider the patient to have “possible” infective endocarditis according to modified Duke criteria (the patient fulfilled three minor criteria: fever, predisposing heart condition, microbiological evidence) [10].

Defervescence was observed after one week of treatment and inflammatory markers normalized. Three blood cultures were sterile. Therapy was continued for a total of four weeks.

At a follow-up visit 6 months after discontinuation of therapy, the patient was doing well and had sterile blood cultures.

### Review of the literature

A review of the literature on endocarditis due to *Granulicatella spp* was performed by a PubMed search for the period between 1997 and 2011 using the following keywords: endocarditis, *Granulicatella adiacens*, *Abiotrophia defectiva*, nutritionally variant Streptococci. A study was considered eligible for inclusion in the review if it reported data on the localization of the disease, isolation of the bacterium, antibiotic treatment (type and, in some cases, duration), and outcome of patients. Our search identified 16 relevant articles, describing a total of 20 patients (fourteen men, five women, one unspecified), with mean age of 47 years (Table 1). The analysis of clinical aspects of these patients revealed that nine had risk factors for IE (such as structural heart disease, recent cardiac surgical procedures), echocardiography was negative for signs of endocarditis in two patient, peripheral stigmata of endocarditis were absent in six and unknown in five. Regarding the treatment, beta-lactam plus gentamicin was the empiric therapeutic regimen in 18/20 patients; in two cases even rifampin was used in the treatment of attack. The treatment duration varied (from 2 to 6 weeks) (in seven cases treatment data were not available). Combined surgical and medical treatment was undertaken in eight patients and in two cases there were relapse after discontinuation of therapy. Christensen et al. [11] reported 3 pediatric cases with IE due to *Granulicatella spp*.

**Table 1:** Case reports of Granulicatella spp. Endocarditis described in the literature

**Author**	**Age (years)**	**Etiology**	**Sex**	**Underlyng condition**	**Blood Culture**	**Ecocardiography Vegetations**	**Peripheral Stigmate of Endocarditis**	**Surgery**	**Regimen**	**Duration**	**Outcome**
Roggenkamp O. et al. (1997)	38	G. adiacens	/	None	Positive	/	Yes	Yes	Pi + T and G then added V	/	No Relapse
Heath et al. (1998)	45	G. adiacens	M	None	Positive	Positive (Transesophageal)	No	/	P + G(4 weeks) then P (2 weeks) then oral Cl (2 weeks)	4weeks + 2weeks + 2weeks	/
Heath et al. (1998)	50	G. adiacens	M	None	Positive	Positive	No	/	P + G(2 weeks) followed by Ce (2 weeks)	2weeks + 2weeks	/
Christensen J. et al. (1999)	71	G. adiacens	F	Artificial aortic valve with ascending aorta prosthesis	Positive	/	No	No	P + G	6 weeks	Recurrence 2 months later needing valve replacement
Rosenthal O. et al. (2002)	68	G. adiacens	M	Pacemaker,Bypass grafting,Carotid endoarterectomy, Prosthetic replacement of the intrarenal aorta, Diabetes	Positive	Positive (transesophageal)	No	No	P + R + G the latter discontinued due to low level resistance	/	No relapse
Casalta J.P. et al. (2002)	29	G. elegans	M	Bicuspit aortic valve	Negative	Positive (both transthoracic and transesophageal)	Yes	Yes	Ax + G	5 weeks	No relapse
Wijetunga et al. 2002	31	A. adiacens	M	None	Positive	Positive	Yes	No	Ce + G	4 weeks	No relapse
Perkins A. et al. (2003)	57	G. adiacens	M	Mitral regurgitation	Positive	Negative (transthoracic)	Yes	No	Ap + G	Ap (4 weeks) G (2 weeks)	No relapse
Ohara-Nemoto Y. et al. (2005)	53	G. elegans	F	None	Positive	/	Yes	Yes	P + G	4 weeks	No relapse
Jeng A. et al. (2005)	18	G. adiacens	M	Congenital heart disease	Positive	Positive (both transthoracic and transesophageal)	Yes	No	V + G + R	6 weeks	No relapse
Al-Tawfiq JA. et al. (2006)	47	G. elegans	M	Mitral valve prolapse	Positive	Positive (both transthoracic and transesophageal	No	Yes	P + Cx	6 weeks	No relapse
Hernando Real S. et al. (2007)	77	G. adiacens	F	Aortic stenosis and mitralic insufficiency	Positive	Positive (transesophageal)	No	No	Ap + G	4 weeks	No relapse
Schwede I. et al. (2007)	41	G. adiacens	M	Connatal aortic stenosis	Positive	Negative	/	/	Ce then P + G	6 weeks	Relapse 6 weeks later
Chang S. et al. (2008)	31	G. adiacens	M	None	Positive	Positive (transthoracic)	Yes	No	Ox + G	/	No relapse
Vandana K. Et al. (2010)	71	G. adiacens	M	None	Positive	Positive	Yes	No	B + G	4 weeks	No relapse
Lin CH et al. 2007	18	G. adiacens	F	None	Positive	Positive	/	Yes	P + G	/	No relapse
Lin CH et al. 2007	61	G. adiacens	M	None	Positive	Positive	/	Yes	P + G	/	No relapse
Lin CH et al. 2007	30	G. adiacens	M	None	Positive	Positive	/	Yes	P + G + Cx	/	No relapse
Lin CH et al. 2007	28	G. adiacens	F	None	Positive	Positive	/	Yes	P + G then V and then Te	/	No relapse
Laho D et al. 2011	81	G. adiacens	M	Aortic disease and mitralic insufficiency	Postivie	Positive (transesophageal)	No	No	Ap + G	Ap (4 weeks) G (2 weeks)	No relapse

M: male; F: female;/: unknown; Pi: piperacillin; T: tazobactam; G: gentamicin; V: vancomicin; P: penicillin; Cl: clindamycin; R: rifampin; Ax: amoxicillin; Ap: ampicillin; Cx: cefotaxime; Ce: ceftriaxone; B: beta-lactam; Ox: oxacillin; Te: teicoplanin.

## Conclusions

We reported two cases of *Granulicatella* bacteremia and IE. Endocarditis involving the valve homograft was demonstrated in one by echocardiography and was strongly suspected in the second case. Both patients failed first line antibiotics, but subsequently responded to a second line regimen including meropenem.

In the adult population, NVS endocarditis has a higher mortality rate (17%), then IE caused by *enterococci* (9%) or *viridans streptococci* (0-12%) [12]. It is not clear if mortality is similarly increased in the pediatric population.

NVS endocarditis may be extremely difficult to treat. In particular, treatment of *Abiotrophia/Granulicatella* infection is complicated by variable susceptibility to commonly used antistreptococcal antibiotics, such as penicillin and ceftriaxone, as well as significant resistance to macrolides [13,14]. Vancomycin demonstrates susceptibility *in vitro*, while gentamicin and rifampicin may be useful for synergy [13]. Nevertheless, treatment failure was observed in about 41% of the cases, despite sensitivity of the organisms to the antibiotics used in two-thirds of these cases [12], and almost 27% of reported cases required prosthetic valve replacement [15]. Moreover, the optimal duration of treatment has not been elucidated.

Since in both our patients failed first line antibiotic treatment was observed, we used a combination of meropenem with another anti-streptococcal drug, based on previous reports suggesting up to 96% of strains may be susceptible to meropenem [16,17]. To our knowledge this is the first description of *Granulicatella spp* bacteraemia successfully treated with a combination therapy including meropenem. Blood cultures quickly became sterile and surgical therapy was not required (conduit obstruction observed in the first case was not clearly a direct consequence of IE, since previous dysfunction had been documented and cultures of the excised graft were sterile). AST of serial isolates from the first patient was obtained and it showed unexpected results. The initial blood culture isolate had the expected patterns of antibiotic sensibility. However, a second isolate, obtained after 5 days of treatment was resistant to cephalosporins, tetracycline, chloramphenicol and linezolid and the MIC for vancomycin had increased to 1.5 mcg/ml, a concentration that may predict a poor *in vivo* response. Of note, to our knowledge this is the first report of a strain of *Granulicatella adiacens* that is resistant to linezolid.

In conclusion, in *Granulicatella* IE in the pediatric population, a combination antibiotic regimen including meropenem should be considered as second line treatment in patients who fail to respond to conventional recommendations. Finally, pediatricians should be aware of the possibility of *Granulicatella* bacteremia and IE in little patients with structural heart disease or prosthetic cardiac valves and those undergoing cardiac procedures.

## Consent

Written informed consents were obtained from both patient’s parents for publication of these case reports. A copy of the written consents is available for review by the Series Editor of this journal.

## Abbreviations

NVS: Nutritionally variant Streptococcus; AST: Antibiotic susceptibility tests; MIC: Minimum inhibitory concentration; IE: Infective endocarditis.

## Competing interests

The authors declare that they have no competing interests.

## Authors’ contributions

Each author has contributed greatly to write this article. MDL and PB have been involved in the data collection and in drifting the manuscript. DA, SC and MAC have made substantial contribute to the review of the literature.GR, FICC and PD’A have been involved in the diagnostic and clinical management of two patients and have given final approval of the version to be published. All authors read and approved the final manuscript.

## Pre-publication history

The pre-publication history for this paper can be accessed here:

http://www.biomedcentral.com/1471-2431/13/61/prepub
